# Ante-mortem characterization of sudden deaths as first-manifestation in Italy

**DOI:** 10.1007/s10840-021-00949-5

**Published:** 2021-02-27

**Authors:** Antonio Frontera, Matteo Anselmino, Mario Matta, Andrea Baccelli, Konstantinos Vlachos, Alessandro Bonsignore, Claudia Camaioni, Pasquale Notarstefano, Saagar Mahida, Martina Nesti, Frederic Sacher, Roberto Tunzi, Giovanni Landoni, Daniela Aschieri, Vincenzo Castelli, Meleze Hocini, Pierre Jaïs, Fiorenzo Gaita, Nicolas Derval, Michel Haïssaguerre

**Affiliations:** 1 Arrhythmology Department, IRCCS San Raffaele Scientific Institute and San Raffaele Hospital, Vita-Salute San Raffaele University, Via Olgettina 60, 20132 Milan, Italy; 2 Electrophysiology Department, LIRYC Institute, Bordeaux University Hospital, Bordeaux, France; 3 Cardiology Division, “Città Della Salute e della Scienza di Torino” Hospital, Department of Medical Sciences, University of Turin, Turin, Italy; 4 Department of Legal and Forensic Medicine, University of Genova, Genoa, Italy; 5 Cardiology Department, Istituto clinico Città Studi, Milan, Italy; 6 Cardiology Department, San Donato Hospital, Arezzo, Italy; 7 Liverpool Centre for Cardiovascular sciences, and Liverpool Heart and Chest Hospital, Liverpool, UK; 8 Cardiology Department, University of Bari, Bari, Italy; 9 Anesthesia and Intensive Care Department, IRCCS San Raffaele Scientific Institute, Vita-Salute San Raffaele University, Milan, Italy; 10 Cardiology Department, Castel San Giovanni Hospital, Piacenza, Italy; 11 Fondazione Giorgio Castelli ONLUS, Rome, Italy

**Keywords:** Sudden death, Young, Cardiopulmonary resuscitation, Idiopathic VF, young adults

## Abstract

**Purpose:**

There is a relative paucity of data on ante-mortem clinical characteristics of young (age 1 to 35 years) sudden death (SD) victims. The aim of the study was to characterize ante-mortem characteristics of SD victims, in a selected national cohort identified by a web search.

**Methods:**

A dataset of all SD (January 2010 and December 2015) was built from national forensic data and medical records, integrated with Google search model. Families were contacted to obtain consent for interviews. Data were obtained on ante-mortem symptoms. ECG characteristics and autopsy data were available.

**Results:**

Out of 301 SD cases collected, medical and family history was available in 132 (43.9%). Twenty-eight (21.1%) had a positive family history for SD. SD occurred during sport/effort in 76 (57.6%). One hundred twelve (85%) SD cases had no prior reported symptoms. Autopsy data were available in 100/132 (75.8%) cases: an extra cardiac cause was identified in 20 (20%). Among the 61 cases with a cardiac diagnosis, 21 (34%) had hypertrophic cardiomyopathy. Among the 19 (19%) victims without structural abnormalities, molecular autopsy identified pathogenic variants for channelopathies in 9 cases. Ten (10%) victims had no identifiable cause.

**Conclusions:**

Most SD were due to cardiac causes and occurred in previously asymptomatic patients. SD events mainly occurred during strenuous activity. In a minority of cases, no cause was identified. The web-based selection criteria, and incomplete data retrieval, need to be carefully taken into account for data interpretation and reproducibility.

**Supplementary Information:**

The online version contains supplementary material available at 10.1007/s10840-021-00949-5.

## Introduction

Sudden death (SD) is a significant public health concern, with particularly devastating consequences in young patients. In cohorts aged 1 to 40 years, the incidence of SD has been reported in the range of 0.9 to 8.5 per 100,000 person-years [1–3], with cardiac disease accounting for the vast majority of non-traumatic cases [4–7].

Data on ante-mortem characteristics of SD events are conflicting. Mellor et al. reported that sudden arrhythmic death more commonly occurs at rest or during sleep and up to 90% of victims do not have prior symptoms or identifiable risk factors for sudden arrhythmic death syndrome (SADS) [8]. However, an autopsy-based series of a US military population (18 years of age and over) undergoing active surveillance reported that among witnessed SD cases, 40% were temporally associated with exertion, and more than half of SD victims were symptomatic within 1 week of the terminal event [9]. These findings suggest that symptoms may manifest weeks before the terminal SD event.

Due to the lack of detailed ante-mortem data from young SD victims, we sought to analyze the ante-mortem characteristics of young adults (< 35 years old) in whom SD was the first clinical manifestation using a nationwide population-based analysis.

## Methods

### Patient cohort

A dataset of young SD victims (age 1 to 35 years) who suffered SD as a first clinical manifestation, between January 1, 2010, and December 31, 2015, was created. In the absence of a national registry on sudden death, the dataset was built based on the national forensic database and medical records integrated with Google search model.

**Fig. 1:**
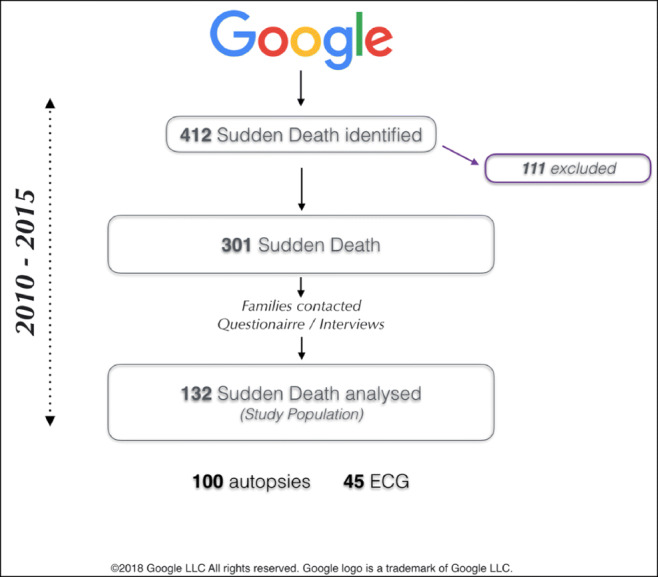
Cases of sudden death. Among the 412 SD identified through google search module, 111 were excluded because were toxicology positive, or were cases of homicide/suicide. Our analysis focused on 301 sudden death: families were contacted and data collected

SD was defined as a non-traumatic, unexpected fatal event occurring within 1 hour from the onset of symptoms in an otherwise healthy subject (if death was not witnessed, the definition was applied when the victim was in good health 24 h before the event). SD was defined as unexplained when no cause was identified after a complete and comprehensive autopsy examination, including histologic and toxicological studies. Patients with known cardiac disease and/or positive toxicology screens at autopsy were excluded (Fig. 1). Initially, 412 SD victims were identified; however, after careful review by two independent researchers (A.F. and M.A.), 111 cases did not satisfy inclusion criteria (see details in Supplemental Figure 1).

### Data collection

The investigators contacted the local hospitals where the SD event was registered. Family members and/or 1st-degree relatives of the SD victims were offered participation to the study and contacted by the investigators to obtain formal consent for interviews. A standardized questionnaire which focused on ante-mortem, as well as post-mortem (autopsy and genetic tests) data, was used (details included in Supplemental data). The questionnaire specifically focused on (1) SD prodrome; (2) past medical history, including congenital abnormalities and abnormal anthropometric measurements; (3) detailed breakdown of sports participation; (4) first assistance given to the victim (e.g., cardiopulmonary resuscitation), presence of on-site medical personnel, basic life support and early defibrillation with an automatic external defibrillator (BLSD), proficiency among rescuers, AED availability, first recorded rhythm at AED arrival, number of shocks. Where available, ECG and autopsy data were collected from the relatives, as well as from forensic and medical records and sport medicine physicians distributed along the Italian territory. Each case was carefully evaluated, and available witnesses were interviewed personally by the investigators. Additionally, primary data were retrieved through the EMS run sheets. Any missing data prevented the enrollment of the patient in the study. Autopsies were performed at the respective centers according to Italian pathologists’ guidelines, recommending collection of histological and toxicological data (including a panel of most commonly abused substances and alcohol). The study protocol was approved by the local ethics committee at the Città della Salute e della Scienza di Torino Hospital.

### Statistics

Continuous variables were expressed as mean ± standard deviation (SD) and categorical variables as number and percentage. A *t* test or a chi square test was performed for comparison of continuous and categorical variables, respectively. *P* values < 0.05 were considered statistically significant. Data were analyzed using SPSS software 20.

### Patient and public involvement

This research was performed with the participation of the victims’ relatives, who represented the largest source of ante-mortem data of the victims. Relatives were not invited to comment on the study design or interpret the results.

## Results

### Study population

**Fig. 2:**
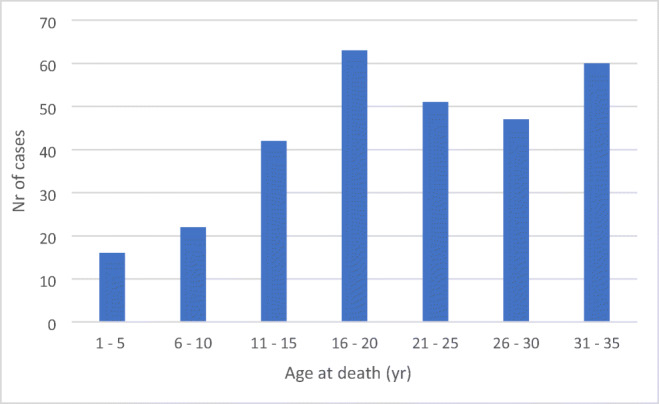
Sudden death according to age group (*N* = 301)

During the study period (from 2010 through 2015), Italy had a population of 60.2 million residents, of whom 21.1 million were aged 1 to 35 years [10]. The number of deaths in this age group during the aforementioned study period was 7304. In the present study, we identified 301 cases of SD, yielding an estimated annual incidence of sudden unexpected death in Italy of 0.24 cases per 100,000 persons (95% confidence interval, 0.13 to 0.45). Males had a higher incidence of SD (0.4 vs. 0.1 cases per 100,000 persons, *P* < 0.001). The majority of the victims were male (81%) and SD occurred at a mean age of 22 ± 7.9 years (Fig. 2). 28/132 (21.1%) cases had a family history of SD (first-degree relatives in 9% of cases; second/third-degree relatives in 12.1%). 101/132 (76.5%) undertook sport; 67 of whom (50.8%) at a high intensity level (Table 1).

**Table 1:** Demographic characteristics of the sudden death cohort

Age, mean (± SD), years	22 ± 7.9
Male, *n* (%)	107 (81%)
Ethnicity, *n* (%)	
Caucasian	126 (95.4%)
African	4 (3.0%)
Hispanic	1 (0.8%)
Asiatic	1 (0.8%)
BMI, mean (± SD)	24 ± 6.5
Distinctive features, *n* (%)	
Multiple nevi	5 (3.8%)
Marfanoid habitus	3 (2.3%)
Pectus excavatum	1 (0.8%)
Pectus carinatum	1 (0.8%)
Region, *n* (%)	
North	35 (26.6%)
Centre	46 (34.8%)
South	51 (38.6%)
Occupational status, *n* (%)	
Student	64 (48.5%)
Employee	17 (12.9%)
Athlete	8 (6.0%)
Other	43 (32.6%)
Sport activity, *n* (%)	
Active (> 5 h/week)	43 (32.6%)
Amateur	34 (25.8%)
None	31 (23.5%)
Competitive	24 (18.2%)

### Ante-mortem characteristics

112/132 (84.8%) victims had no prior reported symptoms, 13 (9.8%) reported intense fatigue in the 2 weeks preceding the SD event, 3 (2.3%) suffered from palpitations in the last 3 months, 2 (1.5%) had a history of syncope, and 2 (1.5%) had a history of pre-syncope. Ten SD victims (7.6%) were on antibiotic therapy at the time of death. Fourteen (10.6%) victims had associated pyrexia in the days before the event (Table 2). One victim, a male professional bodybuilder, reported regular use of anabolic steroids; post-mortem examination revealed a previously undiagnosed hypertrophic cardiomyopathy (with a heart weight of 780 g). Four victims had a prior history of using of protein powder (two underwent post-mortem examination; 1 had a final diagnosis of HCM and 1 of a ruptured cerebral aneurysm (Table 2**)**.

**Table 2:** Clinical and behavioral variables of the sudden death victims

Symptoms before SD, *n* (%)	
No prior symptoms	112 (84.8%)
Fatigue	13 (9.8%)
Palpitations	3 (2.3%)
Syncope	2 (1.5%)
Pre-syncope	2 (1.5%)
Allergies, *n* (%)	9 (6.8%)
Pulmonary disease, *n* (%)	
History of pneumonia	8 (6%)
Asthma	3 (2%)
Tuberculosis	1 (0.8%)
Previous surgery, *n* (%)	19 (14.4%)
History of seizures, *n* (%)	3 (2.3%)
Associated pyrexia, *n* (%)	14 (10.6%)
Recent trauma, *n* (%)	5 (3.8%)
Medical therapy at the time of SD, *n* (%)	
NSAID	4 (3%)
Antibiotics	10 (7.6%)
Oral contraceptives	3 (12%)†
Corticosteroids	2 (1.5%)
Insulin	1 (1%)
Anti-epileptic drugs	1 (1%)
No therapy	111 (84.1%)
Cardiovascular risk factors, *n* (%)	
Smoke	10 (7.6%)
Hypertension	0
Type 1 diabetes mellitus	2 (1.5%)
Drugs addiction, *n* (%)	5 (3.8%)
Energy drinks routine use, *n* (%)	12 (9.1%)
Anabolic steroids, *n* (%)	1 (1%)
Protein Powder, *n* (%)	4 (3%)
Sudden increase in size in the last 3 months (when applicable), n (%)	5 (3.7%)‡

†Among female victims

‡Among victims < 20 years of age

### Circumstances of death

A significantly higher number of SD events occurred in the winter months (*P* = 0.1, Table 3). In 116/132 (87.9%), the SD event occurred in an urban setting. Seventy-eight (57.6%) SD events occurred in the context of high adrenergic tone (sport *n* = 37; physical effort *n* = 31; immediate post-effort recovery *n* = 10). In 5 cases (3.8%), SD took place during intense emotional stress.

**Table 3:** Circumstances of sudden death

Time, *n* (%)	
Morning (6 am–12 pm)	32 (24.2%)
Afternoon/evening (12 pm–11 pm)	71 (53.8%)
Night (11 pm–6 am)	33 (25%)
Season, *n* (%)	
Winter	42 (31.8%)
Spring	33 (25%)
Summer	27 (20.5%)
Autumn	30 (22.7%)
Location, *n* (%)	
Urban	116 (87.9%)
Rural	16 (12.1%)
Witnessed, *n* (%)	101 (76.5%)
SD occurred during, *n* (%)	
Sport/effort/post-effort recovery	76 (57.6%)
Rest/sleep	42 (31.8%)
Emotional stress	5 (3.8%)
Miscellanea	9 (6.8%)
SD took place at	
Sport facility	49 (37.1%)
Home	43 (32.6%)
School	12 (9.1%)
Other (i.e., public streets, bars, beaches etc.)	28 (21.2%)

### Resuscitation data

Among the 101 witnessed SD events, CPR began promptly (within 1 min) in 28 cases (27.7%). Overall, 120/132 (91%) SD cases received CPR. An automatic external defibrillator (AED) was available onsite in 19 cases (14.4%). Forty-five of 120 rescuers (37.5%) were BLS-certified. The first recorded rhythm obtained by AED analysis was available for 101 victims (56 [55.4%] VF; 39 [38.6%] asystole; 6 [6%] pulseless electrical activity). Complete data on the immediate management by bystanders and emergency medical personnel are included in Table 4.

**Table 4:** Resuscitation data

Behavior of the victim, *n* (%)	
Gasping	41 (31%)
Eyes wide open	31 (23.5%)
Seizure-like movements	28 (21.2%)
Urine/stool emission	24 (18.2%)
Vomit	13 (9.8%)
AED available onsite, *n* (%)	19 (14.4%)
AED onsite (only sports centers, *n* = 49)	7 (14.3%)
CPR began promptly (within 1 min), *n* (% out of witnessed SD)	28 (27.7%)
CPR performed by BLS-certified bystander, *n* (% out of total no. of CPRs)	45 (37.5%)
Ventilation performed, *n* (%)	30 (22.7%)
Rhythm at AED arrival, *n* (% among available tracings, *n* = 101)	
VF	56 (55.4%)
ASYSTOLE	39 (38.6%)
PEA	6 (6%)
Shocked at least once, *n* (%)	55 (41.7%)

### Electrocardiographic data

ECGs were available for 45/132 (34%) subjects. In 8% of the cases, ECGs were performed as part of routine pre-participation sports screening. Four cases had incomplete right bundle branch block, nine had early repolarization (inferior with horizontal slope in all cases), one had a short QT interval (QTc 289 msec), and one had QT interval prolongation (QTc 540 msec) (Supplemental Figure 2). The patient with QT interval prolongation was on antibiotic therapy (penicillin).

### Post-mortem characteristics

**Fig. 3:**
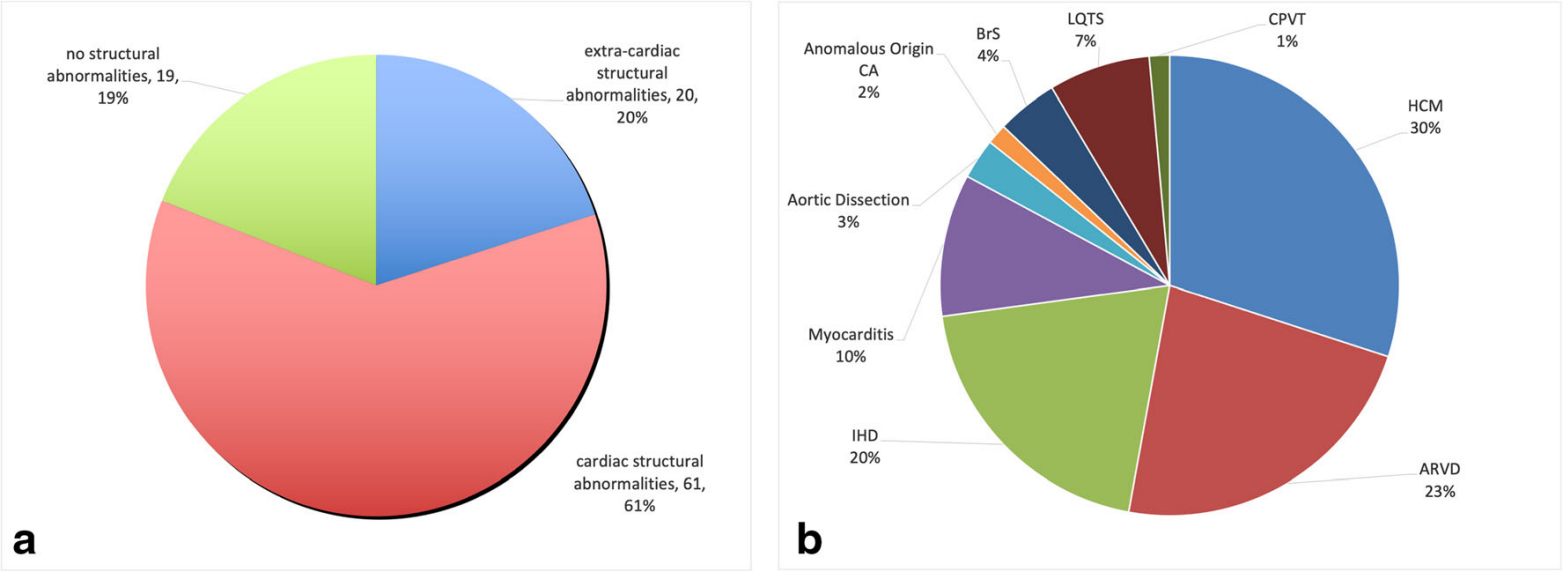
Left and right panel respectively show causes of sudden death and of sudden cardiac death. IHD: ischemic heart disease, HCM: hypertrophic Cardiomyopathy, ARVD: arrhythmogenic right ventricular dysplasia, MYO: myocarditis, BrS: Brugada Syndrome, LQTS: Long QT Syndrome, CPVT: Catecholaminergic Polymorphic Ventricular Tachycardia

Autopsy data were available in 100 cases. Cardiac structural disease accounted for 61 (61%) of all autopsied SD cases. Extracardiac causes were identified in 20 cases (20%). There were no identified structural abnormalities in 19 (19%) of the autopsies (Fig. 3**a**). Among the sudden cardiac deaths, 21/61 (34.4%) had hypertrophic cardiomyopathy (HCM), 16 (26.2%) arrhythmogenic right ventricular cardiomyopathy (ARVC), 14 (23%) ischemic heart disease (IHD), 7 (11.5%), myocarditis, 2 DeBakey type II aortic dissection (3.3%), and 1 (1.6%) an anomalous origin of the coronary arteries (Fig. 3**b**). Extracardiac causes of SD included cerebrovascular events (*n* = 15, 75% among extracardiac SD), possible drowning (*n* = 1), sepsis (*n* = 1), and DeBakey type I and III aortic dissections (*n* = 3). Among the 61 cases presenting structural cardiac disease, the final diagnosis was achieved by gross and histopathologic studies, without genetic testing. Among the 19 victims without signs of macroscopic or microscopic cardiac structural disease, genetic testing for variants implicated in heritable cardiomyopathies and/or channelopathies (long QT syndrome, short QT syndrome, Brugada syndrome (BrS), and catecholaminergic polymorphic ventricular tachycardia) was performed as per consensus guidelines. In the aforementioned 19 cases, molecular autopsy resulted in a diagnosis of Brugada syndrome in 3 cases, long QT syndrome (LQTS) in 5 cases, and catecholaminergic polymorphic ventricular tachycardia (CPVT) in 1 case. The cause of death was not identified in 10 cases (sudden unexplained death).

**Fig. 4:**
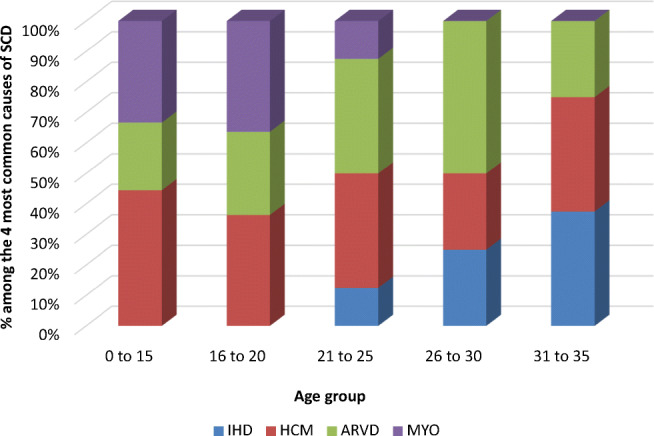
Trends of the 4 most common causes of SCD by age group. IHD, ischemic heart disease; HCM, hypertrophic cardiomyopathy; ARVD, arrhythmogenic right ventricular dysplasia; MYO, myocarditis

The cause of death varied according to age group; considering the four most common causes of SCD, HCM and myocarditis were the most frequent causes of SD in the 16 to 20 age group and ARVC was the most frequent cause of SD in the 26 to 30 age group. The incidence of IHD as a cause of SD increased progressively from the ages of 21 to 35, with no reported cases in the 16 to 20-year age group (Fig. 4).

### Clinical screening among relatives of the victims

Screening data from relatives (parents and siblings) was available for 70 SD cases. Family screening with an ECG or echocardiogram was performed in all these cases. Genetic testing was performed in only 5 cases. A definite clinical diagnosis was established in 6 of the 70 (8.5%) screened families (CPVT, *n* = 1; BrS, *n* = 1; ARVC, *n* = 2; HCM, *n* = 1). There was one diagnosis of aortic stenosis in the mother of a victim. No deaths occurred among relatives of the victims over 41 ± 11 months of follow-up.

## Discussion

In the present study, based on a national cohort identified by a web search, we report ante-mortem and post-mortem characteristics from an SD cohort aged 1–35 years. The findings of our study are as follows: (1) Only 15% of subjects had symptoms before the SD event, with fatigue, palpitations, syncope, and pre-syncope being the most commonly reported symptoms from the patients’ relatives. (2) Close to two-thirds of SD events occurred in the context of high adrenergic tone. (3) The age-related distribution of SD showed a bimodal trend, with the first peak at 15–20 years and a second peak at 30–35 years, with the underlying causes differing significantly between age groups.

Based on our findings, the estimated annual incidence of sudden unexpected death in Italy during the study period was 0.24 per 100,000 persons. A recent nationwide study [11] reported higher estimates of ~ 1.3/100,000-person-years. The differences in the estimates highlight the potential limitations associated with web-based population selection and incomplete data retrieval. Despite these limitations, however, given the paucity of data on ante-mortem clinical characteristics of young SD victims, we believe that the present study provides valuable insights into the terminal event leading to SD. Of note, in the present study, despite attempted resuscitation in almost every victim, there was no return of spontaneous circulation at any point.

The majority of SD events in the present study occurred in the context of activities associated with high adrenergic tone. These findings are in contrast with a recent report from Bagnall et al. [11], where most of the SD events occurred at rest or during sleep. There are several potential explanations for the observed differences between studies. In our study, the leading causes of SD were ARVC, HCM, and IHD, conditions that are typically associated with physical exercise-induced SD. The most frequent causes of death in the aforementioned study by Bagnall et al. were sudden unexplained cardiac death (with an overall diagnostic yield of genetic testing of 27%, mostly for channelopathies), CAD, and DCM. The differences in the underlying causes may account for the differing circumstances of SD events. Another potential explanation is population selection bias, with SD events occurring in the context of sporting activity being more likely to be reported by media and therefore identified by the Google search™ module. In this respect, the abovementioned study by Bagnall and colleagues, in which all cases of sudden death in subjects 1 to 35 years of age were prospectively collected by forensic pathology centers, potentially represents more accurate and representative data.

Concerning acute management of SD events, even though the majority of SD events were witnessed, less than a third received immediate CPR (defined as CPR delivery within 1 min). Furthermore, only a minority of resuscitation providers were BLS trained, and AEDs were available in an even smaller proportion of cases. These findings further underscore the importance of BLSD training campaigns and wider AED availability to improve survival.

SD was associated with a bimodal trend in the present study, with the first peak at 15–20 years and a second peak at 30–35 years of age. The underlying causes of SD were significantly different between age groups, with myocarditis and HCM representing the most common cause in the former age group, and IHD and HCM accounting for more than half of cases in the latter. The most common finding at autopsy was a cardiac structural abnormality, followed by an extracardiac structural pathology. However, a fifth of the cases had no evidence of structural anomalies. Molecular autopsy in the latter group yielded a causative variant in 47% of cases. There findings indicate that the addition of a molecular autopsy significantly increases the likelihood of determining the cause of SD.

### Clinical perspectives

As to whether mass screening programs represent a cost-effective means of preventing SD among children and young adults remains controversial. However, our study shows that a significant proportion of victims had a positive family history for SD, which highlights the importance of a targeted and thorough cardiological and genetic evaluation among first and second-degree relatives. Of note, approximately 40% of sudden cardiac deaths in the young remain unexplained after autopsy. These findings indicate that the addition of molecular autopsy is important in patients with unexplained SD and patients with structurally abnormal hearts, in search of genotype-phenotype associations. Standard molecular autopsy panels will typically include the four main genes accounting for a significant number of previously unexplained SCDs, including *KCNQ1* (LQT1), *KCNH2* (LQT2), *SCN5A* (LQT3/BrS1), and *RYR2* (CPVT1). Finally, the results of our investigation call for further AED diffusion and BLSD training campaigns among the general population.

### Limitations

The present study has a number of limitations. The authors acknowledge the fact that the dataset is not based on a prospective national registry, and variable media coverage, particularly in poorer and minority populations could potentially result in selection bias. Furthermore, only 132 of 301 SD cases were included for analysis, which may further increase bias. Autopsies and genetic testing, when performed, were carried out at different institutions throughout the country, with significant variation in analytical practices. This was not a geographically or ethnically diverse population, and therefore, the results may not be generalizable to other populations. The potential for misclassification of drug overdose-related SD should be acknowledged, as reported by two recent large reports [12, 13]. However, in Italy, the public prosecutor commonly requests complete forensic investigation, including the collection of toxicological data. Therefore, in our cohort, the risk of this type of misclassification is reduced significantly*.*

## Conclusions

The majority of sudden unexpected deaths in patients under 35 years are due to cardiac causes. While cardiomyopathies prevail in younger age, ischemic heart diseases are a more prevalent cause in older patients. The vast majority of SD occur in previously asymptomatic patients, mainly during strenuous activity, further highlighting the importance of exercise screening programs. In 10% of the cases, no clear cause of SD could be identified. Web-based population selection, together with difficulties in complete data retrieval, need to be carefully considered for data interpretation and reproducibility.

## Supplementary information

ESM 1 (PDF 203 kb)
