# Supplementary material for: Ante-mortem characterization of sudden deaths as first-manifestation in Italy
Source: J Interv Card Electrophysiol. 2021 Feb 27;63(2):267–74. doi: 10.1007/s10840-021-00949-5 (PMC8983542; doi:10.1007/s10840-021-00949-5)
Supplement: Supplementary file 1 — (PDF 203 kb) [file 10840_2021_949_MOESM1_ESM.pdf]

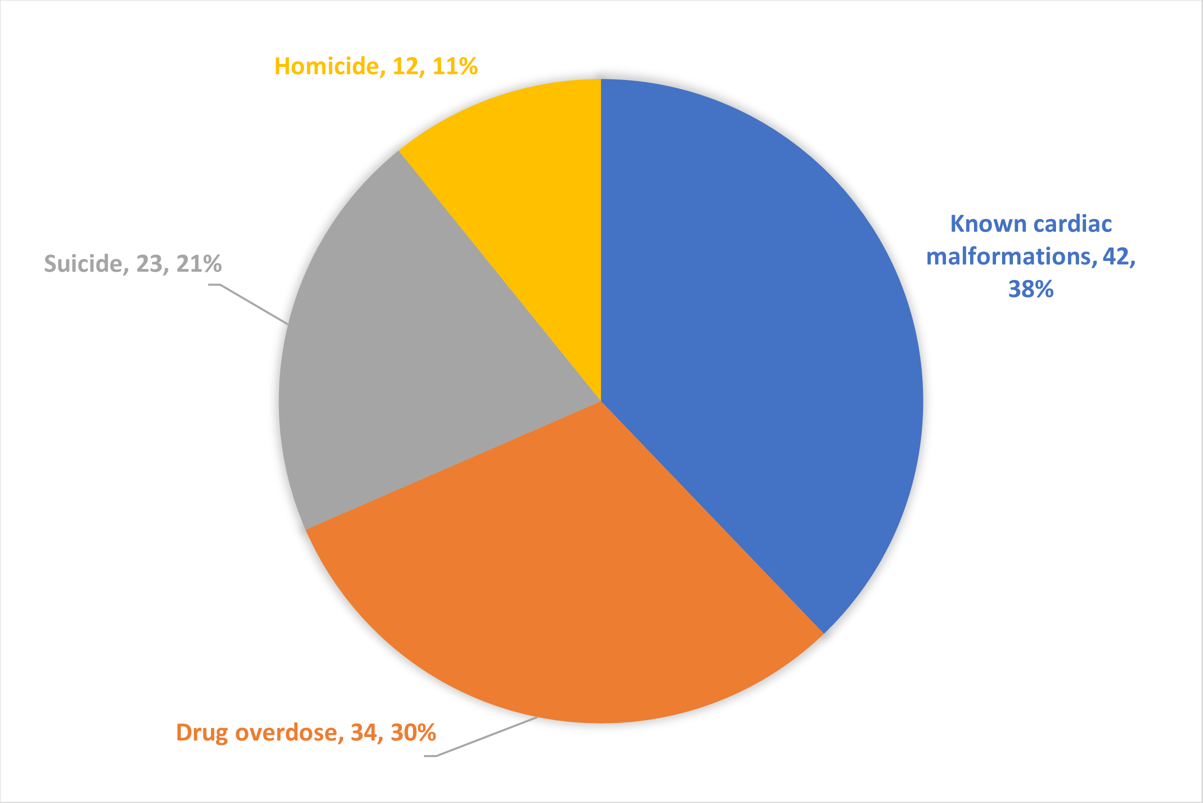

Supplemental figure 1. Excluded SD cases (n = 111)

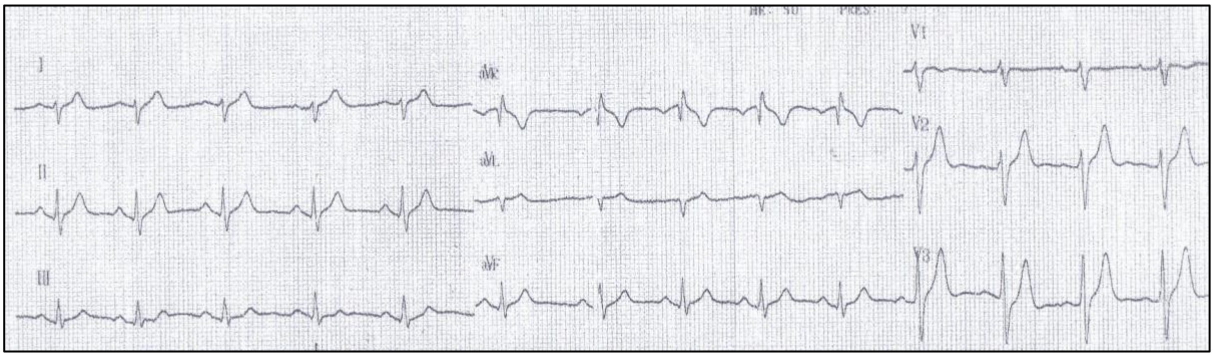

Supplemental figure 2. 12 leads ECG of the victim with short QT interval
